# Supplementary material for: BAMboozle removes genetic variation from human sequence data for open data sharing
Source: Nat Commun. 2021 Oct 28;12:6216. doi: 10.1038/s41467-021-26152-8 (PMC8553849; doi:10.1038/s41467-021-26152-8)

Supplementary Information

BAMboozle removes genetic variation from human sequence data for open data sharing

Christoph Ziegenhain[1] and Rickard Sandberg[1+]

[1]Department of Cell and Molecular Biology, Karolinska Institute, Stockholm, Sweden

[+]Correspondence to: rickard.sandberg@ki.se

Supplementary Figure 1. Transcriptome complexity and copy-number variation analysis. (**a**) Downsampling of sequence reads to evaluate the observed transcriptome complexity (here quantified as the number of detected genes) at fixed numbers of sequence reads per cell. Boxplots show the detected number of genes per cell in the original sequence data and after processing with *BAMboozle*, for each of the five cell lines separately. Unmapped reads were excluded for this analysis, as they are removed during processing with *BAMboozle*. (**b**) Copy-number variations (CNVs) were inferred from original (left) and processed (right) sequence data using the *inferCNV* R-package. One of the cell lines was arbitrarily chosen as the required reference sample in *inferCNV*. Shown is a heatmap of amplified (shades of red) and depleted (shades of blue) genomic regions in each of the cells.

Supplementary Figure 1

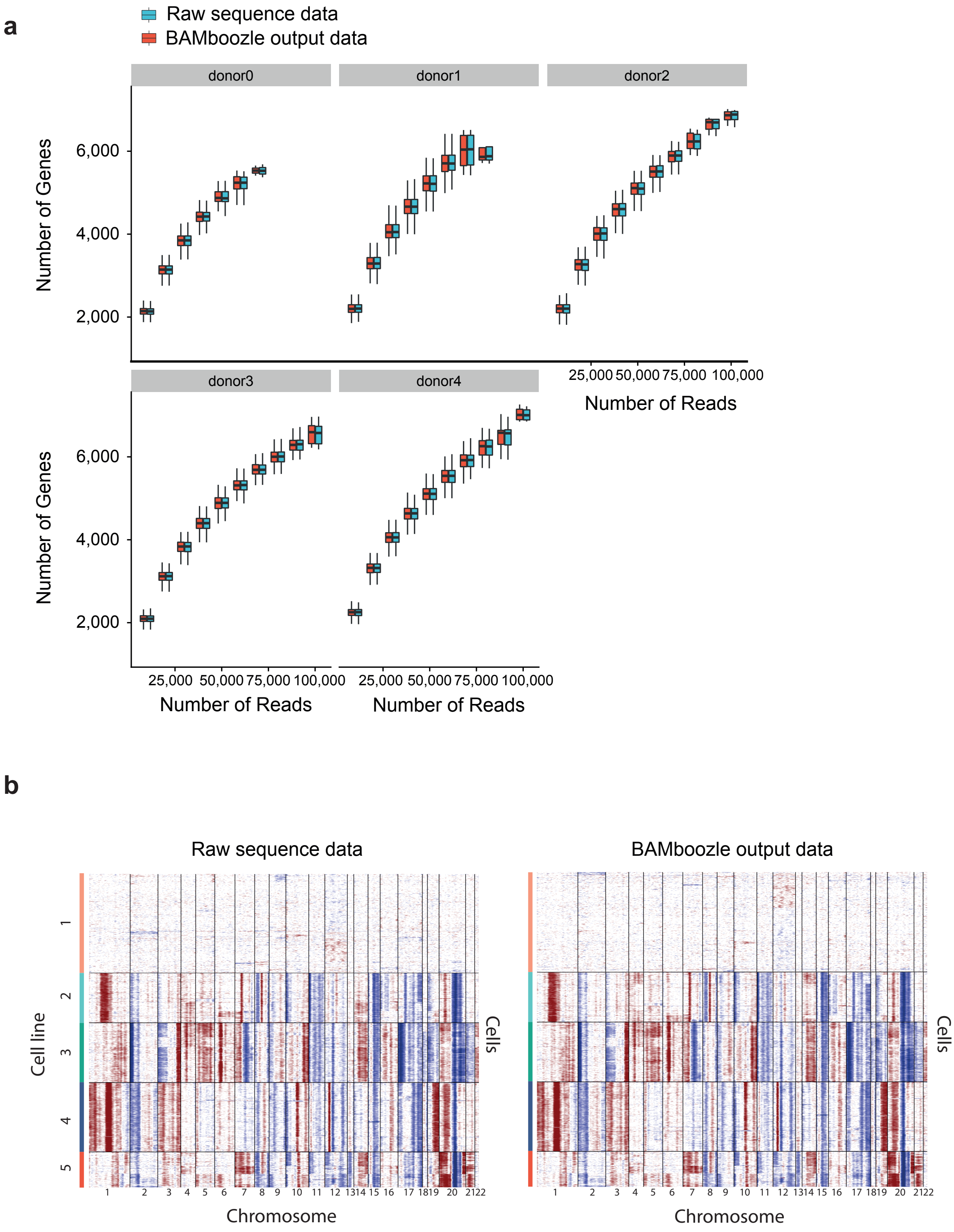

Supplement: Supplementary file 1 — Supplementary Information [file 41467_2021_26152_MOESM1_ESM.pdf]
